# Supplementary material for: Toxicological Evaluation of Kaempferol and Linearolactone as Treatments for Amoebic Liver Abscess Development in Mesocricetus auratus
Source: Int J Mol Sci. 2024 Oct 2;25(19):10633. doi: 10.3390/ijms251910633 (PMC11477209; doi:10.3390/ijms251910633)
Supplement: Supplementary file 1 [file ijms-25-10633-s001.zip › Supplementary Materials 230924.pdf]

International Journal of *Molecular Sciences*

MDPI

Supplementary Materials

Toxicological Evaluation of Kaempferol and Linearolactone as Treatments for Amoebic Liver Abscess Development in *Mesocricetus auratus*

Luis Varela-Rodríguez [1,†], Fernando Calzada [2], José Antonio Velázquez-Domínguez [3,†], Verónica Ivonne Hernández-Ramírez 3, Hugo Varela-Rodríguez [1,4], Elihú Bautista [5], Mayra Herrera-Martínez [6], Diana Laura Pichardo-Hernández [3], Rodrigo Daniel Castellanos-Mijangos [7], Bibiana Chávez-Munguía [3] and Patricia Talamás-Rohana [3,*]

1 Facultad de Ciencias Químicas (FCQ), Universidad Autónoma de Chihuahua (UACH), Chihuahua 31125, CP, Mexico

2 Unidad de Investigación Médica en Farmacología, Hospital de Especialidades UMAE-CMNSXXI-IMSS, Ciudad de México 06725, CP, Mexico

3 Departamento de Infectómica y Patogénesis Molecular, CINVESTAV-IPN, Ciudad de México 07360, CP, Mexico

4 Facultad de Medicina y Ciencias Biomédicas, Universidad Autónoma de Chihuahua (UACH), Chihuahua 31109, CP, Mexico

5 Unidad de Ciencias Ambientales, IPICYT, San Luis Potosí 78216, CP, Mexico

6 Instituto de Farmacobiología, Universidad de la Cañada (UNCA), Teotitlán de Flores Magón, Oax 68540, CP, Mexico

7 Unidad de Imagenología Diagnóstica, Centro Médico ISSEMyM-Arturo Montiel Rojas, Metepec 52170, CP, Mexico

* Correspondence: ptr@cinvestav.mx; Tel.: +52-(55)-5747-3351

† These authors contributed equally to this work and should be considered co-first authors.

Abstract: Several studies with kaempferol (KP) and linearolactone (LL) have demonstrated their antiparasitic activity. However, the toxicity of these treatments is unknown. Therefore, this study aimed to evaluate the possible toxicological effects of intraperitoneal (i.p.) administration of KP or LL on the amoebic liver abscess model (ALA) in *Mesocricetus auratus*. An ALA was induced in male hamsters with 1.5×10^5 *Entamoeba histolytica* (*E. histolytica*) trophozoites inoculated in the left hepatic lobe. The lesion evolved for 4 days, and then KP (5 mg/kg body weight/day) or LL (10 mg/kg body weight/day) was administered for 4 consecutive days. Then, magnetic resonance imaging (MRI), paraclinical analyses, and necropsy for histopathological evaluation were performed. There was similar ALA inhibition by KP (19.42%), LL (28.16%), and metronidazole, the antiamoebic control (20.87%) ($p \leq 0.05$, analysis of variance [ANOVA]). There were hepatic and renal biochemical alterations in all treatment groups, mainly for KP (aspartate aminotransferase: 347.5 ± 37.5 U/L; blood urea nitrogen: 19.4 ± 1.9 g/dL; $p \leq 0.05$, ANOVA). Lesions found in the organs were directly linked to the pathology. In conclusion, KP and LL decreased ALA development and exerted fewer toxicological effects compared with metronidazole. Therefore, both compounds exhibit therapeutic potential as an alternative treatment of amoebiasis caused by *E. histolytica*. However, additional clinical studies in different contexts are required to reaffirm this assertion.

Keywords: amoebic liver abscess; *Entamoeba histolytica*; kaempferol; linearolactone; *Mesocricetus auratus*; toxicity

Publisher's Note: MDPI stays neutral about jurisdictional claims in published maps and institutional affiliations.

Copyright: © 2021 by the authors. Licensee MDPI, Basel, Switzerland. This article is an open access article distributed under the terms and conditions of the Creative Commons Attribution (CC BY) license (https://creativecommons.org/licenses/by/4.0/).

1. Supplementary Figures

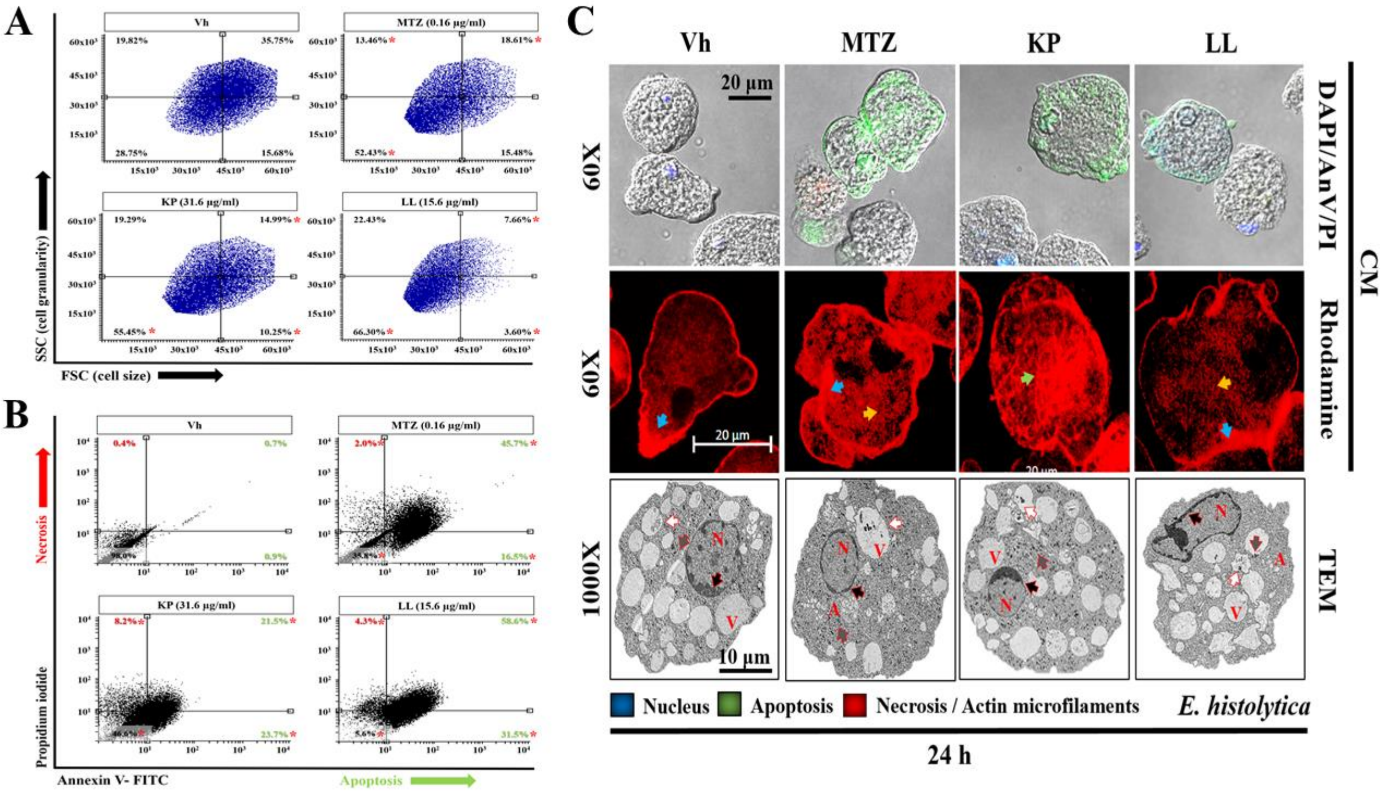

Figure S1. Biological activity of KP and LL in trophozoites. (**A**) Morphological changes, such as size or granularity, were observed. (**B**) Trophozoites (1 x10^5) were washed twice with 1X PBS, stained with AnV/PI kit (Biovin, K109-100) and processed to read 20 x10^3 events in three independent analyzes by flow cytometry. (**C**) The morphology of trophozoites was confirmed by Confocal Microscopy (CM) (LSM-700, Zeiss®) and Transmission Electron Microscopy (TEM) (JEM-1011, JEOL®) under similar conditions. The arrows indicate actin microfilaments (green), polymeric actin (yellow), cortical actin (blue), euchromatin (black), glycogen granules (gray), and membrane structures (white). Results show the mean ± SD of three biological replicates ($n = 3$, in triplicates). (*) $p \leq 0.05$ vs. vehicle (1X PBS, negative control), ANOVA. Metronidazole was used as positive control (antiparasitic reference).

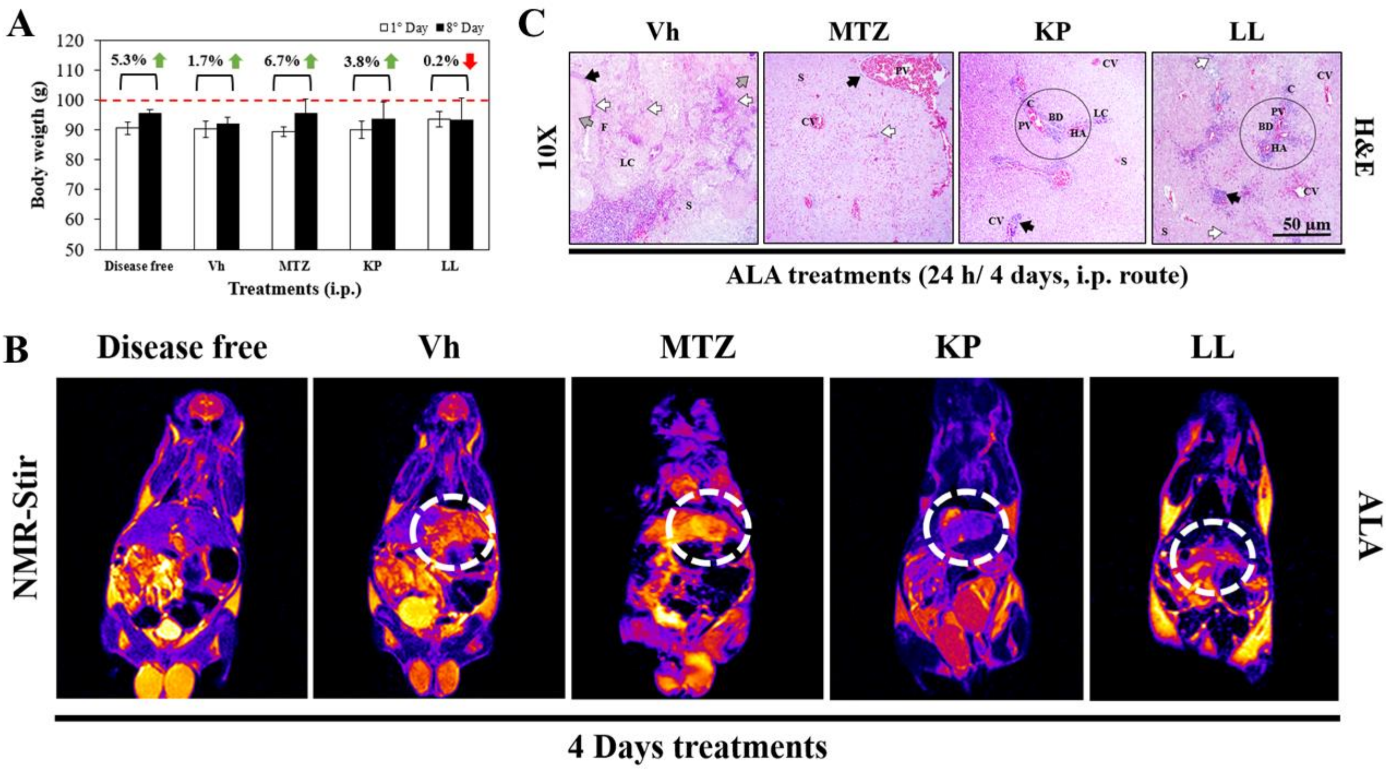

Figure S2. Antiamoebic activity of KP and LL against ALA. (**A**) The body weight of the hamsters was monitored with an electronic scale every 24 h after infection with *E. histolytica* trophozoites during the 8-day period of the trial. Treatments were administered i.p. 4 to 8 days after infection, the animals were subsequently necropsied. The dotted line delimits the section made at 100 g of hamster body weight. Green and red arrows represent body weight gain (↑) and loss (↓), respectively. (**B**) An NMR imaging study was performed to observe the densitometric and morphological changes in the ALA, as well as to rule out the rupture of the ALA and the subsequent migration process of amoebic trophozoites that could compromise other structures and areas surrounding the hepatic region, such as the diaphragm (up to the lungs and heart) or the peritoneal cavity (later systemic invasion). Liver lesions are outlined with a white circle. (**C**) Histopathological studies using microscopic slides and H&E staining revealed relevant changes in the typical architecture of liver tissue during treatment with KP (5 mg/kg body weight/24 h) or LL (10 mg/kg body weight /24 h). The following figures show hepatic lobes with centrilobular veins (CV) and portal triads (circles) at the apices; a portal vein (PV); a hepatic artery (HA); and a bile duct (BD) with cholangioles (C); numerous lymphatic capillaries (LC) and sinusoids (S); a fibrous liver stroma and a parenchyma with numerous typical hepatocytes; Kupffer cells and abundant leukocyte infiltrates (black arrows); and abundant tissue necrosis due to a proinflammatory response (gray arrows) to *E. histolytica* trophozoite infection (white arrows). Data are presented as means ± SD (n = 5). ANOVA. *$p \leq 0.05$

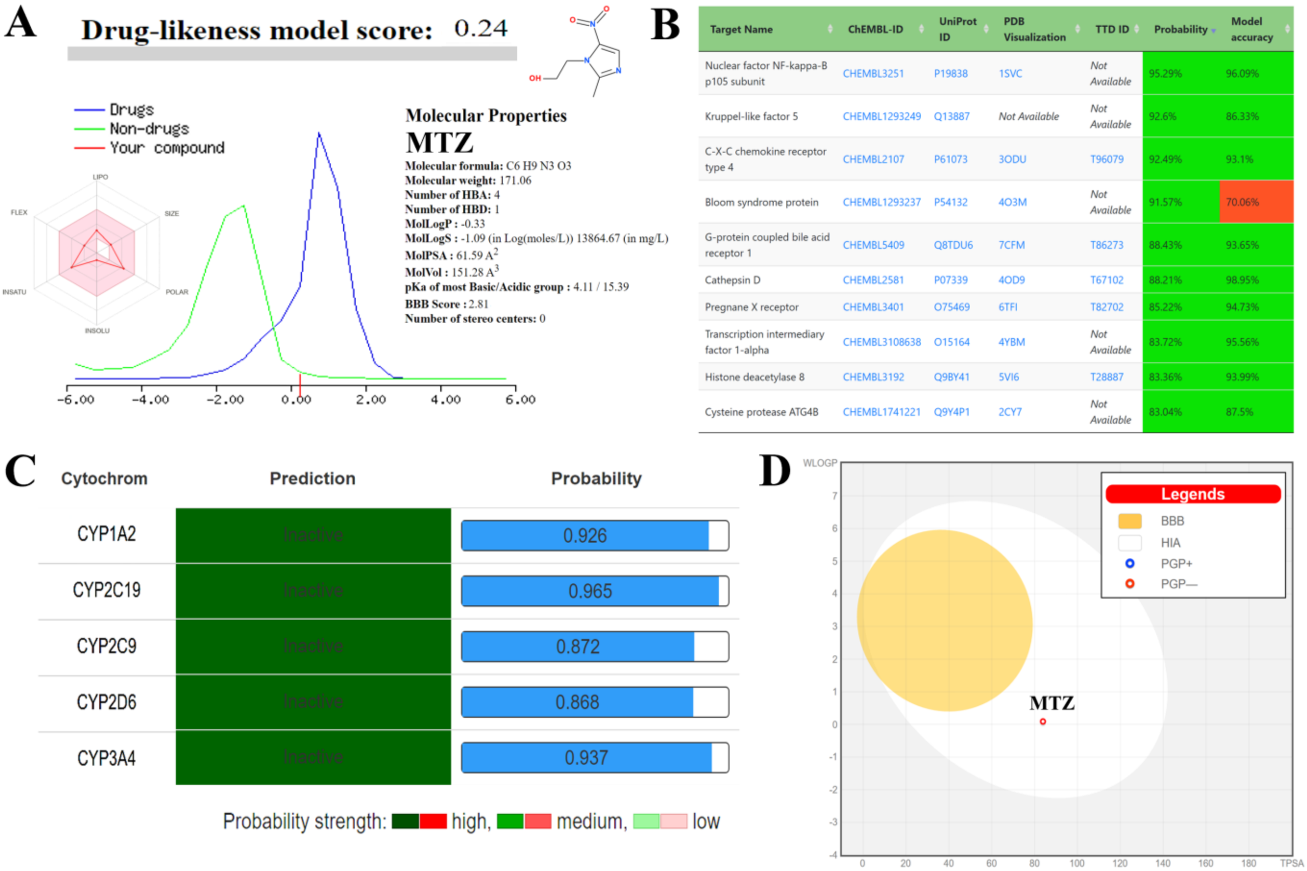

Figure S3. Predictions about pharmacokinetic, toxicity and molecular targets, based on physicochemical properties for MTZ. Molecular properties and chemical structure of MTZ were used to predict activity through the Drug-likeness model of Molsoft© (**a**) and their possible target pharmacophores by SwissTarget Prediction of SIB© (**b**). The toxicological activity of MTZ like antiparasitic drug was predicted through inhibitory effects on the enzymatic complex associated with CYP by a machine learning predictive model based on specific fingerprints called MCF with SuperCYPsPred of Structural Bioinformatics© (**c**). Other toxicity features such as permeability to BBB, HIA, and affinity to PGP were predicted through BOILED-Egg permeation method by SwissADME of SIB© (**d**). Abbreviations used: MTZ, metronidazole; MCF, most common features; BBB, blood-brain barrier; HIA, health impact assessment; PGP, P-glycoprotein; BOILED, Brain Or IntestinaL EstimateD; ADME, absorption, distribution, metabolism and excretion; CYP, cytochrome-P450 system; HBA, hydrogen bond acceptors; HBD, hydrogen bond donors; Log *P*, partition coefficient; Log *S*, aqueous solubility coefficient; pK_a, acid dissociation coefficient; PSA, polar surface area; LIPO, lipophilicity; POLAR, polarity; INSOLU, insolubility; INSATU, insaturation; FLEX, flexibility.

2. Supplementary Tables

Table S1. Paraclinical studies in hamsters with ALA treated with KP and LL.

Treatments	Disease-free	Vh	MTZ	KP	LL	Reference
Glucose (mg/dL)	116 ± 13	144 ± 36	161 ± 21	152 ± 11	123 ± 20	65 – 176 (89)
Triglycerides (mg/dL)	260 ± 41 *	378 ± 49 *	280 ± 75	475 ± 28 *	132 ± 8.7 *	30 – 145 (88)
Cholesterol (mg/dL)	93 ± 7.8 *	163 ± 21 *	116 ± 9.1	164 ± 43 *	105 ± 24 *	94 – 237 (166)
Total protein (g/dL)	5.6 ± 0.1 *	4.2 ± 0.2 *	5.3 ± 0.9 *	5.2 ± 0.2	5.3 ± 0.3	4 – 8.6 (6.2)
**Hepatic parameters**						
GGT (U/L)	5.5 ± 0.7	9 ± 3	5.5 ± 2.3	9.3 ± 5.5	7.5 ± 2.1	1 – 10 (5.5)
Albumin (g/dL)	1.9 ± 0.1	1.6 ± 0.2	1.9 ± 0.3	1.7 ± 0.2	1.7 ± 0.1	2 – 4 (3)
Alkaline phosphatase (U/L)	147 ± 20 *	572 ± 26 *	230 ± 11 *	349 ± 38 *	355 ± 9.5 *	8 – 202 (105)
AST (GOT) (U/L)	79 ± 12 *	158 ± 53 *	81 ± 24 *	59 ± 67 *	163 ± 22 *	55 – 250 (153)
ALT (GPT) (U/L)	64 ± 6.1 *	261 ± 35 *	135 ± 23 *	60 ± 10 *	89 ± 16 *	28 – 107 (68)
Total bilirubin (mg/dL)	0.8 ± 0.1	0.8 ± 0.2	0.7 ± 0.3	0.38 ± 0.07 *	0.37 ± 0.04 *	0.2 – 1 (0.6)
Direct bilirubin (mg/dL)	0.7 ± 0.1	0.63 ± 0.2	0.5 ± 0.1	0.37 ± 0.07	0.26 ± 0.04	0.04 – 0.3 (0.09)
Indirect bilirubin (mg/dL)	0.1 ± 0.0	0.14 ± 0.1	0.20 ± 0.1	0.01 ± 0.0	0.12 ± 0.0	0.16 – 0.7 (0.25)
**Renal parameters**						
Creatinine (mg/dL)	0.6 ± 0.1	0.84 ± 0.2	1.01 ± 0.2 *	0.45 ± 0.1	0.54 ± 0.2	0.2 – 0.9 (0.5)
Urea (mg/dL)	61 ± 2.9	15.2 ± 2.5 *	41.2 ± 1.4	41.6 ± 4	37 ± 6.01	47 – 73 (60)
BUN (g/dL)	19 ± 1.4 *	6.5 ± 0.5 *	19.3 ± 0.7 *	19.4 ± 1.9 *	16.1 ± 1.5 *	11 – 27 (19)
**Hematological parameters**						
Hemoglobin (g/dL)	17 ± 0.3	16. 4 ± 0.3	14. 7 ± 1.2	16.5 ± 0.2	16 ± 0.8	13 – 19 (16)
Hematocrit (%)	50 ± 0.5 *	25 ± 5.3 *	35 ± 2.6 *	48.8 ± 0.5 *	45.4 ± 2.9 *	39-49 (40)
Erythrocytes (x10⁶/mm³)	6.9 ± 0.4 *	3.5 ± 0.4 *	3.7 ± 0.2 *	6.8 ± 0.3 *	6.7 ± 0.4 *	3 – 12 (8)
MCHC (g/dL)	34 ± 0.3 *	60.8 ± 7.6 *	43 ± 1.4 *	33 ± 0.1 *	35 ± 0.9 *	30 – 38 (34)
MCH (pg)	26 ± 0.6 *	48 ± 4.2 *	40 ± 1.6 *	24 ± 0.7 *	24 ± 0.3 *	20 – 26 (32.4)
MCV (fL)	76 ± 1.4	79 ± 2.9	94 ± 2.2 *	72 ± 2.3 *	68 ± 2.2 *	65 – 78 (77.8)
Leukocytes (x100/mm³)	8 ± 0.5	19.6 ± 4.5	7.7 ± 2.6 *	14.8 ± 7	7.1 ± 1.8 *	5 – 12 (8.5)
RDW-CV (fL)	13.4 ± 0.2 *	36.4 ± 0.9 *	25.8 ± 3.3 *	14.3 ± 0.1 *	14.7 ± 0.6 *	--
RDW-SD (fL)	42.5 ± 0.2 *	120 ± 5.7 *	111 ± 12 *	42.4 ± 0.3 *	40 ± 2.3 *	--
Platelets (x10⁶/µL)	291 ± 23	475 ± 143	536 ± 148	537 ± 266	303 ± 31	200 – 500 (350)
MPV (fL)	7.3 ± 0.1 *	9.6 ± 0.7 *	8 ± 0.1 *	7.25 ± 0.5 *	7.7 ± 0.5 *	--
PDW (fL)	7.1 ± 0.7 *	9.7 ± 0.9 *	11 ± 0.4 *	9.15 ± 1.2	9.4 ± 0.3 *	--
PCT (%)	0.2 ± 0.0	0.4 ± 0.2	0.4 ± 0.1	0.4 ± 0.2	0.2 ± 0.0	--

Results show the mean ± S.D. of two biological replicates (*n* = 5).
*, $p \leq 0.05$ vs. the control groups by ANOVA (red for disease-free; green for vehicle; and yellow for both).
Controls used in the study were metronidazole as positive control, vehicle as negative control (1X PBS, with-out treatment), and disease-free as normal control (without ALA).
Reference range, minimum and maximum normal value for the analyte of interest in hamster and the respective mid-range [27,90].
Abbreviations used: KP, kaempferol; LL, linearolactone; MTZ, metronidazole; ALA, amoebic liver abscess; ALT, alanine aminotransferase; AST, aspartate aminotransferase; GGT, gamma glutamyl-transpeptidase; BUN, blood urea nitrogen; PCT, plateletcrit; MCHC, mean corpuscular hemoglobin concentration; MCH, mean corpuscular hemoglobin; MCV, mean cell volume; RDW-CV, red cell blood distribution width-volume; RDW-SD, red cell blood distribution width-size; MPV, mean platelet volume; PDW, platelet distribution width.

Table S2. Bioinformatic analysis of possible pharmacophore targets to KP.

Target	Common name	Uniprot ID	ChEMBL ID	Target Class	Probability*	Known actives (3D/2D)
NADPH oxidase 4	NOX4	Q9NPH5	CHEMBL1250375	Enzyme	1.0	7 / 8
Aldose reductase (by homology)	AKR1B1	P15121	CHEMBL1900	Enzyme	1.0	18 / 71
Xanthine dehydrogenase	XDH	P47989	CHEMBL1929	Oxidoreductase	1.0	12 / 20
Tyrosinase	TYR	P14679	CHEMBL1973	Oxidoreductase	1.0	2 / 3
Tyrosine-protein kinase receptor FLT3	FLT3	P36888	CHEMBL1974	Kinase	1.0	5 / 7
Carbonic anhydrase II	CA2	P00918	CHEMBL205	Lyase	1.0	8 / 14
Arachidonate 5-lipoxygenase	ALOX5	P09917	CHEMBL215	Oxidoreductase	1.0	5 / 47
Carbonic anhydrase VII	CA7	P43166	CHEMBL2326	Lyase	1.0	8 / 13
Estradiol 17-beta-dehydrogenase 2	HSD17B2	P37059	CHEMBL2789	Enzyme	1.0	9 / 3
Multidrug resistance-associated protein 1	ABCC1	P33527	CHEMBL3004	Primary active transporter	1.0	7 / 11
Estradiol 17-beta-dehydrogenase 1	HSD17B1	P14061	CHEMBL3181	Enzyme	1.0	9 / 4
Aryl hydrocarbon receptor	AHR	P35869	CHEMBL3201	Transcription factor	1.0	1 / 1
Carbonic anhydrase XII	CA12	O43570	CHEMBL3242	Lyase	1.0	10 / 17
Estrogen-related receptor alpha	ESRRA	P11474	CHEMBL3429	Nuclear receptor	1.0	2 / 2
P-glycoprotein 1	ABCB1	P08183	CHEMBL4302	Primary active transporter	1.0	12 / 48
Cytochrome P450 1B1	CYP1B1	Q16678	CHEMBL4878	Cytochrome P450	1.0	12 / 46
ATP-binding cassette sub-family G member 2	ABCG2	Q9UNQ0	CHEMBL5393	Primary active transporter	1.0	6 / 47
Adenosine A1 receptor (by homology)	ADORA1	P30542	CHEMBL226	Family A G protein-coupled receptor	0.79504726301	6 / 23
Carbonic anhydrase IV	CA4	P22748	CHEMBL3729	Lyase	0.79504726301	7 / 12
Acetylcholinesterase	ACHE	P22303	CHEMBL220	Hydrolase	0.76846920113	4 / 26
Monoamine oxidase A	MAOA	P21397	CHEMBL1951	Oxidoreductase	0.65801973764	5 / 18
Glyoxalase I	GLO1	Q04760	CHEMBL2424	Enzyme	0.65801973764	3 / 4
Tyrosine-protein kinase SYK	SYK	P43405	CHEMBL2599	Kinase	0.65801973764	3 / 3
Glycogen synthase kinase-3 beta	GSK3B	P49841	CHEMBL262	Kinase	0.65801973764	3 / 6
Matrix metalloproteinase 9	MMP9	P14780	CHEMBL321	Protease	0.65801973764	2 / 2

Matrix metalloproteinase 2	MMP2	P08253	CHEMBL333	Protease	0.65801973764	2 / 2
Arachidonate 15-lipoxygenase	ALOX15	P16050	CHEMBL2903	Enzyme	0.64978075048	6 / 8
Arachidonate 12-lipoxygenase	ALOX12	P18054	CHEMBL3687	Enzyme	0.63332626242	9 / 10
Receptor-type tyrosine-protein phosphatase S	PTPRS	Q13332	CHEMBL2396508	Phosphatase	0.60844626435	7 / 8
Adenosine A2a receptor (by homology)	ADORA2A	P29274	CHEMBL251	Family A G protein-coupled receptor	0.56730189987	5 / 11
Cyclin-dependent kinase 5/CDK5 activator 1	CDK5R1 CDK5	Q15078 Q00535	CHEMBL1907600	Kinase	0.51792149842	6 / 18
Cyclin-dependent kinase 1/cyclin B	CCNB3 CDK1 CCNB1 CCNB2	Q8WWL7 P06493 P14635 O95067	CHEMBL2094127	Other cytosolic protein	0.51792149842	4 / 11
Arginase-1 (by homology)	ARG1	P05089	CHEMBL1075097	Enzyme	0.51792149842	2 / 2
G-protein coupled receptor 35	GPR35	Q9HC97	CHEMBL1293267	Family A G protein-coupled receptor	0.50153001777	2 / 4
Estrogen receptor beta	ESR2	Q92731	CHEMBL242	Nuclear receptor	0.50153001777	93 / 38
Death-associated protein kinase 1	DAPK1	P53355	CHEMBL2558	Kinase	0.50153001777	2 / 2
DNA-3-methyladenine glycosylase	MPG	P29372	CHEMBL3396943	Enzyme	0.50153001777	1 / 1
Solute carrier family 22 member 12	SLC22A12	Q96S37	CHEMBL6120	Electrochemical transporter	0.50153001777	6 / 1
Transthyretin	TTR	P02766	CHEMBL3194	Secreted protein	0.48489636268	2 / 3
Aldo-keto reductase family 1 member B10	AKR1B10	O60218	CHEMBL5983	Enzyme	0.48489636268	2 / 3
Tankyrase-2	TNKS2	Q9H2K2	CHEMBL6154	Enzyme	0.48489636268	4 / 12
Tankyrase-1	TNKS	O95271	CHEMBL6164	Enzyme	0.48489636268	4 / 28
Cyclin-dependent kinase 6	CDK6	Q00534	CHEMBL2508	Kinase	0.47683561827	3 / 4
Cyclin-dependent kinase 2	CDK2	P24941	CHEMBL301	Kinase	0.47683561827	1 / 17
Cytochrome P450 19A1	CYP19A1	P11511	CHEMBL1978	Cytochrome P450	0.42720527460	6 / 18
Casein kinase II alpha	CSNK2A1	P68400	CHEMBL3629	Kinase	0.42720527460	3 / 2
Epidermal growth factor receptor erbB1	EGFR	P00533	CHEMBL203	Kinase	0.40264337958	7 / 29
Vasopressin V2 receptor	AVPR2	P30518	CHEMBL1790	Family A G protein-coupled receptor	0.40264337958	1 / 1

Insulin-like growth factor I receptor	IGF1R	P08069	CHEMBL1957	Kinase	0.40264337958	3 / 3
Thrombin	F2	P00734	CHEMBL204	Protease	0.40264337958	11 / 3
Serine/threonine-protein kinase PIM1	PIM1	P11309	CHEMBL2147	Kinase	0.40264337958	8 / 7
Serine/threonine-protein kinase Aurora-B	AURKB	Q96GD4	CHEMBL2185	Kinase	0.40264337958	3 / 4
Dopamine D4 receptor	DRD4	P21917	CHEMBL219	Family A G protein-coupled receptor	0.40264337958	1 / 1
Myeloperoxidase	MPO	P05164	CHEMBL2439	Enzyme	0.40264337958	1 / 1
PI3-kinase p85-alpha subunit	PIK3R1	P27986	CHEMBL2506	Enzyme	0.40264337958	1 / 1
Liver glycogen phosphorylase	PYGL	P06737	CHEMBL2568	Enzyme	0.40264337958	1 / 1
Carbonic anhydrase I	CA1	P00915	CHEMBL261	Lyase	0.40264337958	4 / 5
Tyrosine-protein kinase SRC	SRC	P12931	CHEMBL267	Kinase	0.40264337958	3 / 10
Focal adhesion kinase 1	PTK2	Q05397	CHEMBL2695	Kinase	0.40264337958	1 / 2
Vascular endothelial growth factor receptor 2	KDR	P35968	CHEMBL279	Kinase	0.40264337958	2 / 3
Matrix metalloproteinase 13	MMP13	P45452	CHEMBL280	Protease	0.40264337958	1 / 1
Matrix metalloproteinase 3	MMP3	P08254	CHEMBL283	Protease	0.40264337958	1 / 1
Carbonic anhydrase III	CA3	P07451	CHEMBL2885	Lyase	0.40264337958	1 / 1
Serine/threonine-protein kinase PLK1	PLK1	P53350	CHEMBL3024	Kinase	0.40264337958	2 / 3
Carbonic anhydrase VI	CA6	P23280	CHEMBL3025	Lyase	0.40264337958	1 / 1
Cyclin-dependent kinase 1	CDK1	P06493	CHEMBL308	Kinase	0.40264337958	3 / 13
Protein kinase N1	PKN1	Q16512	CHEMBL3384	Kinase	0.40264337958	1 / 3
Carbonic anhydrase XIV	CA14	Q9ULX7	CHEMBL3510	Lyase	0.40264337958	1 / 1
Carbonic anhydrase IX	CA9	Q16790	CHEMBL3594	Lyase	0.40264337958	3 / 6
Hepatocyte growth factor receptor	MET	P08581	CHEMBL3717	Kinase	0.40264337958	4 / 4
Serine/threonine-protein kinase NEK2	NEK2	P51955	CHEMBL3835	Kinase	0.40264337958	1 / 2
Interleukin-8 receptor A	CXCR1	P25024	CHEMBL4029	Family A G protein-coupled receptor	0.40264337958	1 / 1
CaM kinase II beta	CAMK2B	Q13554	CHEMBL4121	Kinase	0.40264337958	1 / 2
ALK tyrosine kinase receptor	ALK	Q9UM73	CHEMBL4247	Kinase	0.40264337958	2 / 4

Serine/threonine-protein kinase AKT	AKT1	P31749	CHEMBL4282	Kinase	0.40264337958	1 / 4
Serine/threonine-protein kinase NEK6	NEK6	Q9HC98	CHEMBL4309	Kinase	0.40264337958	1 / 2
Phospholipase A2 group 1B	PLA2G1B	P04054	CHEMBL4426	Enzyme	0.40264337958	1 / 1
Carbonic anhydrase VA	CA5A	P35218	CHEMBL4789	Lyase	0.40264337958	1 / 1
Beta-secretase 1	BACE1	P56817	CHEMBL4822	Protease	0.40264337958	6 / 17
Tyrosine-protein kinase receptor UFO	AXL	P30530	CHEMBL4895	Kinase	0.40264337958	2 / 4
NUAK family SNF1-like kinase 1	NUAK1	O60285	CHEMBL5784	Kinase	0.40264337958	1 / 2
Aldo-keto reductase family 1 member C2 (by homology)	AKR1C2	P52895	CHEMBL5847	Enzyme	0.40264337958	1 / 1
Aldo-keto reductase family 1 member C1 (by homology)	AKR1C1	Q04828	CHEMBL5905	Enzyme	0.40264337958	1 / 1
Aldo-keto-reductase family 1 member C3 (by homology)	AKR1C3	P42330	CHEMBL4681	Enzyme	0.40264337958	1 / 1
Aldo-keto reductase family 1 member C4 (by homology)	AKR1C4	P17516	CHEMBL4999	Enzyme	0.40264337958	1 / 1
Carbonic anhydrase XIII (by homology)	CA13	Q8N1Q1	CHEMBL3912	Lyase	0.40264337958	1 / 1
Aldehyde reductase (by homology)	AKR1A1	P14550	CHEMBL2246	Enzyme	0.40264337958	1 / 1
Beta amyloid A4 protein	APP	P05067	CHEMBL2487	Membrane receptor	0.29545330488	2 / 12
Poly [ADP-ribose] polymerase-1	PARP1	P09874	CHEMBL3105	Enzyme	0.29545330488	3 / 9
Matrix metalloproteinase 12	MMP12	P39900	CHEMBL4393	Protease	0.29545330488	1 / 1
Lymphocyte differentiation antigen CD38	CD38	P28907	CHEMBL4660	Enzyme	0.29545330488	2 / 2
DNA topoisomerase I (by homology)	TOP1	P11387	CHEMBL1781	Isomerase	0.29545330488	1 / 1
Estrogen receptor alpha	ESR1	P03372	CHEMBL206	Nuclear receptor	0.27083692342	73 / 38
Cyclooxygenase-2	PTGS2	P35354	CHEMBL230	Oxidoreductase	0.27083692342	1 / 25

Cystic fibrosis transmembrane conductance regulator	CFTR	P13569	CHEMBL4051	Other ion channel	0.27083692342	1 / 1
6-phosphofructo-2-kinase/fructose-2,6-bisphosphatase 3	PFKFB3	Q16875	CHEMBL2331053	Enzyme	0.26258150584	2 / 2
AMY1C	AMY1A	P04745	CHEMBL2478	Enzyme	0.23788516757	1 / 1
G protein-coupled receptor kinase 6	GRK6	P43250	CHEMBL6144	Kinase	0.23788516757	2 / 4
Telomerase reverse transcriptase	TERT	O14746	CHEMBL2916	Enzyme	0.22968569932	9 / 22
Microtubule-associated protein tau	MAPT	P10636	CHEMBL1293224	Unclassified protein	0.17197858941	1 / 1

Possible target pharmacophores were predicted through SwissTarget Prediction of SIB© (http://www.swisstargetprediction.ch/).

Table S3. Bioinformatic analysis of possible pharmacophore targets to LL.

Target	Common name	Uniprot ID	ChEMBL ID	Target Class	Probability*	Known actives (3D/2D)
Kappa Opioid receptor	OPRK1	P41145	CHEMBL237	Family A G protein-coupled receptor	0.09723998876	62 /189
Delta opioid receptor	OPRD1	P41143	CHEMBL236	Family A G protein-coupled receptor	0.09723998876	12/ 32
Tankyrase-2	TNKS2	Q9H2K2	CHEMBL6154	Enzyme	0.09723998876	14/ 0
Melatonin receptor 1A	MTNR1A	P48039	CHEMBL1945	Family A G protein-coupled receptor	0.09723998876	99/ 0
Serotonin transporter	SLC6A4	P31645	CHEMBL228	Electrochemical transporter	0.09723998876	41 / 0
Dopamine transporter	SLC6A3	Q01959	CHEMBL238	Electrochemical transporter	0.09723998876	83/ 0
P2X purinoceptor 7	P2RX7	Q99572	CHEMBL4805	Ligand-gated ion channel	0.09723998876	70 / 0
Prostaglandin E synthase	PTGES	O14684	CHEMBL5658	Enzyme	0.09723998876	20 / 0
Melatonin receptor 1B	MTNR1B	P49286	CHEMBL1946	Family A G protein-coupled receptor	0.09723998876	80 / 0
Serotonin 2a (5- HT2a) receptor (by homology)	HTR2A	P28223	CHEMBL224	Family A G protein-coupled receptor	0.09723998876	33 / 0
Serotonin 2c (5- HT2c) receptor	HTR2C	P28335	CHEMBL225	Family A G protein-coupled receptor	0.09723998876	14 / 0
Cyclin-dependent kinase 5/CDK5 activator 1	CDK5R1 CDK5	Q15078 Q00535	CHEMBL1907600	Kinase	0.09723998876	27 / 0
6-phosphofructo-2-kinase/ fructose-2,6-bisphosphatase 3	PFKFB3	Q16875	CHEMBL2331053	Enzyme	0.09723998876	65 / 0
Adenosine A2a receptor	ADORA2A	P29274	CHEMBL251	Family A G protein-coupled receptor	0.09723998876	57 / 0
Adenosine A2b receptor	ADORA2B	P29275	CHEMBL255	Family A G protein-coupled receptor	0.09723998876	49 / 0
Cathepsin K	CTSK	P43235	CHEMBL268	Protease	0.09723998876	40 / 0
Cathepsin S	CTSS	P25774	CHEMBL2954	Protease	0.09723998876	83 / 0
Cytochrome P450 19A1	CYP19A1	P11511	CHEMBL1978	Cytochrome P450	0.09723998876	112 / 0
Estrogen receptor beta	ESR2	Q92731	CHEMBL242	Nuclear receptor	0.09723998876	30 / 0
Bromodomain-containing protein 4	BRD4	O60885	CHEMBL1163125	Reader	0.09723998876	66 / 0
Bromodomain-containing protein 2	BRD2	P25440	CHEMBL1293289	Reader	0.09723998876	36 / 0
Androgen Receptor (by homology)	AR	P10275	CHEMBL1871	Nuclear receptor	0.09723998876	151 / 0

Bromodomain-containing protein 3	BRD3	Q15059	CHEMBL1795186	Reader	0.09723998876	35 / 0
Protein arginine N-Methyltransferase 3	PRMT3	O60678	CHEMBL5891	Writer	0.09723998876	6 / 0
Mu opioid receptor	OPRM1	P35372	CHEMBL233	Family A G protein-coupled receptor	0.09723998876	16 / 55
Cytochrome P450 11B1	CYP11B1	P15538	CHEMBL1908	Cytochrome P450	0.09723998876	17 / 0
Cytochrome P450 11B2	CYP11B2	P19099	CHEMBL2722	Cytochrome P450	0.09723998876	16 / 0
11-beta- Hydroxysteroid dehydrogenase 1	HSD11B1	P28845	CHEMBL4235	Enzyme	0.09723998876	131 / 0
Arachidonate 5-lipoxygenase	ALOX5	P09917	CHEMBL215	Oxidoreductase	0.09723998876	46 / 0
Cannabinoid receptor 2	CNR2	P34972	CHEMBL253	Family A G protein-coupled receptor	0.09723998876	172 / 0
Translocator protein (by homology)	TSPO	P30536	CHEMBL5742	Membrane receptor	0.09723998876	7 / 0
Histamine H3 receptor	HRH3	Q9Y5N1	CHEMBL264	Family A G protein-coupled receptor	0.09723998876	10 / 0
GABA-A receptor; alpha-5/beta-3/ gamma-2	GABRB3 GABRG2 GABRA5	P28472 P18507 P31644	CHEMBL2094122	Ligand-gated ion channel	0.09723998876	9 / 0
Matrix Metalloproteinase 13	MMP13	P45452	CHEMBL280	Protease	0.09723998876	1 / 0
Aldo-keto- reductase family 1 member C3	AKR1C3	P42330	CHEMBL4681	Enzyme	0.09723998876	24 / 0
Rap guanine Nucleotide exchange factor 4	RAPGEF4	Q8WZA2	CHEMBL2029198	Unclassified protein	0.09723998876	2 / 0
Alpha-1a adrenergic receptor	ADRA1A	P35348	CHEMBL229	Family A G protein-coupled receptor	0.09723998876	6 / 0
Hormone sensitive lipase	LIPE	Q05469	CHEMBL3590	Enzyme	0.09723998876	15 / 0
Steroid 5-alpha- reductase 1	SRD5A1	P18405	CHEMBL1787	Oxidoreductase	0.09723998876	4 / 0
Rho-associated protein kinase 2	ROCK2	O75116	CHEMBL2973	Kinase	0.09723998876	4 / 0
Beta-secretase 1	BACE1	P56817	CHEMBL4822	Protease	0.09723998876	45 / 0
Glycine transporter 1	SLC6A9	P48067	CHEMBL2337	Electrochemical transporter	0.09723998876	4 / 0
LDL-associated phospholipase A2	PLA2G7	Q13093	CHEMBL3514	Enzyme	0.09723998876	6 / 0
GABA receptor alpha-1 subunit	GABRA1	P14867	CHEMBL1962	Ligand-gated ion channel	0.09723998876	1 / 0

GABA A receptor alpha-4/beta-3/ gamma-2	GABRB3 GABRA4 GABRG2	P28472 P48169 P18507	CHEMBL2111366	Ligand-gated ion channel	0.09723998876	1 / 0
Thrombin and coagulation factor X	F10	P00742	CHEMBL244	Protease	0.09723998876	42 / 0
GABA receptor alpha-6 subunit	GABRA6	Q16445	CHEMBL2579	Ligand-gated ion channel	0.09723998876	1 / 0
Phosphodiesterase 10A	PDE10A	Q9Y233	CHEMBL4409	Phosphodiesterase	0.0972399887	146 /0
GABA receptor alpha-5 subunit	GABRA5	P31644	CHEMBL5112	Ligand-gated ion channel	0.09723998876	8 / 0
Epoxide hydrolase 1 (by homology)	EPHX1	P07099	CHEMBL1968	Protease	0.0972399887	15 / 0
Liver glycogen phosphorylase	PYGL	P06737	CHEMBL2568	Enzyme	0.09723998876	27 / 0
Dual specificity protein kinase CLK4	CLK4	Q9HAZ1	CHEMBL4203	Kinase	0.09723998876	12 / 0
DNA-dependent protein kinase	PRKDC	P78527	CHEMBL3142	Kinase	0.09723998876	59 / 0
Metabotropic glutamate receptor 5	GRM5	P41594	CHEMBL3227	Family C G protein-coupled receptor	0.09723998876	33 / 0
Serine/threonineprotein kinase AKT	AKT1	P31749	CHEMBL4282	Kinase	0.09723998876	108 / 0
Lysosomal acid lipase/cholesteryl ester hydrolase	LIPA	P38571	CHEMBL4184	Enzyme	0.09723998876	5 / 0
Phosphodiesterase 4D	PDE4D	Q08499	CHEMBL288	Phosphodiesterase	0.09723998876	14 / 0
Epoxide hydratase	EPHX2	P34913	CHEMBL2409	Protease	0.09723998876	93 / 0
Phosphodiesterase 3	PDE3A	Q14432	CHEMBL241	Phosphodiesterase	0.09723998876	2 / 0
Phosphodiesterase 3B	PDE3B	Q13370	CHEMBL290	Phosphodiesterase	0.09723998876	2 / 0
PI3-kinase p110-delta subunit	PIK3CD	O00329	CHEMBL3130	Enzyme	0.09723998876	130 / 0
PI3-kinase p110-beta subunit	PIK3CB	P42338	CHEMBL3145	Enzyme	0.09723998876	147 / 0
PI3-kinase p110-alpha subunit	PIK3CA	P42336	CHEMBL4005	Enzyme	0.09723998876	135 / 0
Bombesin receptor subtype-3	BRS3	P32247	CHEMBL4080	Family A G protein-coupled receptor	0.09723998876	17 / 0
CREB-binding protein/p53	CREBBP	Q92793	CHEMBL5747	Writer	0.09723998876	7 / 0
Transforming	RHOA	P61586	CHEMBL6052	Unclassified	0.09723998876	3 / 0

protein RhoA				protein		
c-Jun N-terminal kinase 3	MAPK10	P53779	CHEMBL2637	Kinase	0.09723998876	14 / 0
Sodium channel protein type IX alpha subunit	SCN9A	Q15858	CHEMBL4296	Voltage-gated ion channel	0.09723998876	284 / 0
Cannabinoid receptor 1	CNR1	P21554	CHEMBL218	Family A G protein-coupled receptor	0.09723998876	136 / 0
MAP kinaseactivated Protein kinase 2	MAPKAPK 2	P49137	CHEMBL2208	Kinase	0.09723998876	3 / 0
Cyclin-dependent kinase 1	CDK1	P06493	CHEMBL308	Kinase	0.09723998876	6 / 0
PI3-kinase p110-gamma subunit	PIK3CG	P48736	CHEMBL3267	Enzyme	0.09723998876	62 / 0
Polyadenylatebinding protein 1	PABPC1	P11940	CHEMBL 1293286	Unclassified	0.09723998876	10 / 0
Histone deacetylase 2	HDAC2	Q92769	CHEMBL1937	Eraser	0.09723998876	6 / 0
Mineralocorticoid receptor	NR3C2	P08235	CHEMBL1994	Nuclear receptor	0.09723998876	25 / 0
Glucocorticoid receptor	NR3C1	P04150	CHEMBL2034	Nuclear receptor	0.09723998876	50 / 0
Progesterone receptor	PGR	P06401	CHEMBL208	Nuclear receptor	0.0972399887	67 / 0
Glutamate [NMDA] receptor subunit epsilon 2	GRIN2B	Q13224	CHEMBL1904	Ligand-gated ion channel	0.09723998876	1 / 0
Dopamine D4 receptor	DRD4	P21917	CHEMBL219	Family A G proteincoupled receptor	0.09723998876	30 / 0
Quinone reductase 2	NQO2	P16083	CHEMBL3959	Enzyme	0.09723998876	10 / 0
Voltage-gated potassium channel subunit Kv1.5	KCNA5	P22460	CHEMBL4306	Voltage-gated ion channel	0.09723998876	29 / 0
Thromboxane-A synthase	TBXAS1	P24557	CHEMBL1835	Cytochrome P450	0.09723998876	7 / 0
Phosphodiesterase 4B	PDE4B	Q07343	CHEMBL275	Phosphodiesterase	0.09723998876	18 / 0
Phosphodiesterase 7A	PDE7A	Q13946	CHEMBL3012	Phosphodiesterase	0.09723998876	7 / 0
Autotaxin	ENPP2	Q13822	CHEMBL3691	Enzyme	0.09723998876	1 / 0
Cyclooxygenase-1	PTGS1	P23219	CHEMBL221	Oxidoreductase	0.0	8 / 0
Cyclooxygenase-2	PTGS2	P35354	CHEMBL230	Oxidoreductase	0.0	31 / 0

Glutathione transferase omega 1	GSTO1	P78417	CHEMBL3174	Enzyme	0.0	2 / 0
Platelet-derived growth factor receptor beta	PDGFRB	P09619	CHEMBL1913	Kinase	0.0	4 / 0
Inosine-5'-monophosphate dehydrogenase 2	IMPDH2	P12268	CHEMBL2002	Oxidoreductase	0.0	28 / 0
Epidermal growth factor receptor erbB1	EGFR	P00533	CHEMBL203	Kinase	0.0	75 / 0
Thrombin	F2	P00734	CHEMBL204	Protease	0.0	6 / 0
Voltage-gated potassium channel, KQT	KCNQ2	O43526	CHEMBL2476	Voltage-gated ion channel	0.0	2 / 0
Vascular endothelial growth factor receptor 2	KDR	P35968	CHEMBL279	Kinase	0.0	97 / 0
Protein kinase C delta	PRKCD	Q05655	CHEMBL2996	Kinase	0.0	8 / 0
Poly [ADP-ribose] polymerase-1	PARP1	P09874	CHEMBL3105	Enzyme	0.0	31 / 0
Protein kinase C theta	PRKCQ	Q04759	CHEMBL3920	Kinase	0.0	10 / 0
Monoamine oxidase A	MAOA	P21397	CHEMBL1951	Oxidoreductase	0.0	5 / 0
Dual-specificity tyrosinephosphorylation regulated kinase 1A	DYRK1A	Q13627	CHEMBL2292	Kinase	0.0	9 / 0
Urotensin II receptor	UTS2R	Q9UKP6	CHEMBL3764	Family A G protein-coupled receptor	0.0	5 / 0

Possible target pharmacophores were predicted through SwissTarget Prediction of SIB© (http://www.swisstargetprediction.ch/).
